# Assessing the Readability, Quality, and Reliability of Online Patient Information Materials Related to Sarcopenia

**DOI:** 10.7759/cureus.99993

**Published:** 2025-12-24

**Authors:** Gozde Bumin Aydin, Tugba Kaya Kurten, Ismail Senih AKSU, Volkan Hancı

**Affiliations:** 1 Anesthesiology and Critical Care, Sincan Training and Research Hospital, Ankara, TUR; 2 Anesthesiology and Critical Care, Dokuz Eylul University Medical Faculty Hospital, Izmir, TUR

**Keywords:** patient education materials, quality, readability, reliability, sarcopenia

## Abstract

Objective: This study aimed to evaluate the readability, quality, and reliability of online patient education materials (PEM) related to sarcopenia.

Materials and methods: In August 2024, 278 websites obtained by searching the term ‘sarcopenia’ on Google were evaluated. After applying the exclusion criteria, 66 websites were included in the study. The ranks of the websites were evaluated with Blexb (New York, USA), readability evaluations were done with calculators, and quality and reliability were evaluated per the Journal of the American Medical Association (JAMA), Global Quality Scale (GQS), and modified Decision Index for Systematic Consumer Evaluation of Reviewed Narratives (DISCERN) criteria.

Results: The readability values of the contents were compared with the 6th-grade level (GRL), and all results were significantly higher than the 6th GRL (p<0.01). The median values in the readability assessment of the 66 websites included in the study were as follows: Flesch Reading Ease Score (FRES) 40.81 (6.10-72.84), Gunning Fog (GFOG) 14.78 (9.10-22.20), Flesch-Kincaid Grade Level (FKGL) 12.18 (7.16-18.58), Coleman-Liau (CL) score 12.89 (7.14-18.45), Simple Measure of Gobbledygook (SMOG) 12.34 (7.77-17.65), Automated Readability Index (ARI) 12.49 (7.70-19.64), Linsear Write (LW) 13.30 (6.89-25.05), and GRL 15.25 (8.00-23.00). The types of websites were compared in terms of readability levels, and no significant difference was found. When the website typologies were compared, no significant difference was observed in GQS, JAMA, and DISCERN. A low-level negative correlation was found between Blexb rank and JAMA. The correlations between Blexb rank and readability scales were as follows: weak negative correlation with FRES and ARI scores and weak positive correlation with GFOG, FKGL, CL, and SMOG scores (p<0.05).

Conclusion: The best category in terms of readability, reliability, and quality was determined to be health portals, but none of the websites had readability at or below the recommended 6th GRL.

## Introduction

Skeletal muscle accounts for 40% of the overall weight of our body and is involved in dynamic functions and metabolic processes. Sarcopenia is a syndrome with a decline in skeletal muscle mass and strength. As the population is aging worldwide, physical inactivity, inadequate nutrient intake, diseases, hormonal factors, and chronic inflammation with decreasing muscle mass and strength trouble society and the healthcare system [1]. The prevalence of sarcopenia is 6% to 12% globally, 14% to 33% in adults aged 65 years and older, and 78% among hospitalized patients [2]. Sarcopenia increases complications after surgery, the risk of hospitalization, debility, mortality, and health care costs [1].

The internet is a popular and accessible tool universally used to get information about health problems. Readability, the property of written material, is important for the reader to understand the content. Most studies showed that the readability of patient education materials (PEM) should not be higher than the 6th- to 8th-grade level (GRL) [3].

Approximately 61% of Americans use the internet to find information related to their health. There is a significant amount of health-related information on the internet due to this demand. There is a necessity for reliable information to be easily found and written at an understandable level that people can rely on in their health decisions [4]. Health literacy is defined as “the degree to which individuals have the ability to find, understand, and use information and services to inform health-related decisions and actions for themselves and others” [4]. Around 22% of Americans have basic health literacy levels, and 14% have below basic health literacy levels, and the average grade reading level (GRL) of Americans is between the 7th and 8th GRL [1].

We hypothesized that most online sarcopenia materials are written for above the 8th GRL and have low quality scores. Most studies recommend that the information should be written two GRLs below the average GRL (e.g., a 6th GRL or below) to be understandable [5]. As the number of words increases in the sentence, readability decreases [5]. The primary objective of this study is to assess online information materials about sarcopenia to determine whether they are at the recommended readability level (6th grade). The secondary outcome is to evaluate these materials in terms of reliability and quality, examining website types, content scope, and variations across search rankings.

## Materials and methods

On August 15, 2024, two independent authors (İ.S.A. and G.B.A.) searched for the exact term "sarcopenia" via the Google search engine (https://www.google.com). A total of 278 websites were retrieved from the search. To mitigate potential inter-rater variability that may arise during evaluation, a third independent author (V.H.) was consulted in cases of disagreement. Since we obtained all the information for this cross-sectional study from publicly available sources and did not conduct any study on individuals or animal subjects, no ethical approval was required.

According to the latest search engine market share values published in July 2023, Google ranks first among search engines with 83.49%, which is why we conducted this study via Google. To ensure that the results obtained were not affected, we deleted the Google search history and all cookies on the computer where the search was made, logged out of all accounts, and searched on Google in incognito mode. We recorded the URLs of a total of 278 websites and evaluated the first 10 results as the 10 most visited sites [6].

Website selection and exclusion criteria

We excluded websites that were scientific studies; used languages other than English; those that were inaccessible, repetitive, did not provide information, contained only videos, provided information on topics unrelated to sarcopenia, made sales, contained articles shorter than 100 words; and YouTube links. Per the exclusion criteria, we included 66 of the 278 website links in our study. We excluded all non-textual content (videos, images, tables, etc.), website links, references, and author information on the websites included in the study and conducted the readability review [6].

Website typologies and ranks

We divided the websites into six different groups by evaluating their purposes, URL extensions (edu., gov., com., org.) and the organizations that own them: non-profit (websites established by non-profit organizations), health-portal (websites established to provide health information), commercial (websites containing product placement for profit), news (websites containing news), government (websites established by governments) and university (websites established by universities) [6]. The Blexb chrome extension (https://www.blexb.com/) provides a dynamic ranking system that tracks user interactions, measuring the frequency and engagement of clicks on different domains. By leveraging this data, websites are systematically ordered according to their Blexb rank, a metric reflecting their relative popularity and user preference within the test group. We used Blexb to evaluate website ranks for an objective, data-driven assessment of web traffic patterns, ensuring an unbiased evaluation of site visibility and user engagement.

Reliability assessment

We used the Journal of the American Medical Association (JAMA) criteria for the reliability of websites. The JAMA criteria include author identities and qualifications, attribution of sources, disclosure of potential conflicts (funding, sponsorship, advertising), and up-to-date information. The included articles received 0 or 1 points for each item [7]. We considered articles with a JAMA score of 0, 1, and 2 as low reliability, and articles with a score of 3 and 4 as high reliability [8].

Quality assessment

We used the Global Quality Scale (GQS) [9] and the modified Decision Index for Systematic Consumer Evaluation of Reviewed Narratives (DISCERN) to evaluate the quality of websites [10]. The GQS is a scoring system consisting of the following items: "Excellent quality and flow, very useful for patients", "Good quality and flow, useful for patients", "Moderate quality, suboptimal flow, somewhat useful for patients", "Very limited use for patients, generally poor quality and poor flow", and "Poor quality, poor flow, not useful for patients at all". The scores range between 1 and 5. Furthermore, per the GQS, we accepted 4 points and above as high quality, 3 points as medium quality, and 2 points and below as low quality [9]. The modified DISCERN is a scoring system that includes 5 questions and is calculated by giving 0 or 1 points for each question depending on the status of meeting these questions [10].

In this study, two independent authors (İ.S.A. and T.K.K.) performed the quality and reliability assessments (JAMA, modified DISCERN, and GQS). The final score used for each website was the average of the two independent authors’ evaluations to enhance objectivity and control for inter-rater variability [11]. Inter-rater reliability was assessed using the interclass correlation coefficient (ICC), which demonstrated high agreement for all subjective measures (ICC > 0.85 for JAMA, DISCERN, and GQS).

Readability assessment

We used two different, free, online calculators to determine the readability levels of websites: Readability Formulas (https://readabilityformulas.com/readability-scoring-system.php) and Online Utility (https://www.online- utility.org/english/readability_test_and_improve.jsp). Using both calculators, we obtained the readability scores of the PEM on each website separately according to different readability formulas. The score values for the same readability formula obtained from both websites were added and divided by two to obtain the readability score value for the relevant website and the PEM. The readability scores evaluated in our study were Flesch Reading Ease Score (FRES), Gunning Fog (GFOG) [12], Flesch-Kincaid Grade Level (FKGL)[13], Coleman-Liau (CL) score [14], Simple Measure of Gobbledygook (SMOG) [15], Automated Readability Index (ARI) [16], and Linsear Write (LW) [17] scoring systems. For text processing, we copied the URLs and contents of the obtained websites and saved them in Word 2023 (Microsoft Corp., Redmond, WA, USA). All non-textual content (videos, images, tables, website links, references, and author information) was excluded from the text. The remaining content was converted to plain text format, punctuation marks other than periods were removed, and evaluated with the specified calculator websites [18]. Since it reflects the average education level, the 6th GRL is the readability level recommended by the American Medical Association (AMA) and the National Institutes of Health (NIH), and therefore, we used this level as the basis for our study.

Content and statistical analysis

We evaluated the content of the websites according to whether they included information on the definition of sarcopenia, pathophysiology, epidemiology, risk factors, symptoms, tests, diagnostic methods, treatment methods, screening scores, complications, effects on morbidity, effects on mortality, protection methods, and prognosis [6]. The data were analyzed using SPSS Statistics version 24.0 (IBM Corp., Armonk, NY, USA). We expressed categorical data as numbers and percentages, and continuous data as median and minimum-maximum. We used the chi-square test and Fisher's sharpness test for the analysis of categorical data, and the Kruskal-Wallis test [19], Mann-Whitney U test [20], and Wilcoxon test [21] in the analysis of continuous data. Spearman's rho correlation analysis was used to evaluate the relationships between Blexb rank, readability, and quality scores. We accepted a p-value below 0.05 as a significant difference.

## Results

Of the 278 websites obtained as a result of our Google search, 66 websites were included in the study based on the inclusion criteria. Of the 212 websites not included in the study, 39.6% were scientific studies, 26.4% were non-textual/YouTube and other video-only content, 18.4% were non-English languages, 10.8% were irrelevant information, 2.4% were advertisements and did not include information, 1.4% were repetition sites, and 0.9% were unreachable websites. Of the 66 websites that met the inclusion criteria, 31.8% were health-related, 15.2% were society/professional organizations, 13.6% were commercial, 12.1% were hospital websites, 9.1% were news websites, 4.5% were government websites, and 3% were physician websites (Table 1).

**Table 1:** Comparison of website typologies and JAMA, modified DISCERN, and GQS scores A p-value <0.05 was considered statistically significant. JAMA: Journal of the American Medical Association, DISCERN: Decision Index for Systematic Consumer Evaluation of Reviewed Narratives, GQS: Global Quality Scale

Parameters		Physician	Society/Professional organization	Health-related website	News	Commercial	Government	Hospital	Other	p-value
Number and percentage		2 (3%)	10 (15.2%)	21 (31.8%)	6 (9.1%)	9 (13.6%)	3 (4.5%)	8 (12.1%)	7 (10.6%)	
JAMA	Insufficient data n:20	0 (0%)	3 (30.0%)	5 (23.8%)	1 (16.7%)	5 (55.6%)	1 (33.3%)	5 (63.5%)	0 (0%)	0.375
	Partially sufficient data n:44	2 (100%)	7 (70%)	15 (71.4%)	5 (83.3%)	4 (44.4%)	2 (66.7%)	3 (37.5%)	6 (85.7%)	
	Completely sufficient data n:2	0 (0%)	0 (0%)	1 (4.8%)	0 (0%)	0 (0%)	0 (0%)	0 (0%)	1 (14.4%)	
Modified DISCERN	Very poor (1.00) n:8	0 (0%)	1 (10%)	3 (14%)	0 (0%)	2 (22.5%)	0 (0%)	2 (25%)	0 (0%)	0.646
	Poor (2.00) n:22	1 (50%)	3 (30%)	3 (14.3%)	3 (50%)	4 (44.4%)	1 (33%)	4 (50%)	3 (42.9%)	
	Fair (3.00) n:22	1 (50%)	3 (30%)	11 (52.4%)	1 (16.7%)	1 (11.1%)	2 (66.7%)	1 (12.5%)	2 (28.6%)	
	Good (4.00) n:9	0 (0%)	2 (20%)	3 (14.3%)	0 (0%)	2 (22.2%)	0 (0%)	1 (12.5%)	1 (14.3%)	
	Excellent (5.00) n:5	0 (0%)	1 (10%)	1 (4.8%)	2 (33.3%)	0 (0%)	0 (0%)	0 (0%)	1 (14.3%)	
GQS	Low quality (1.00-2.00) n:18	0 (0%)	3 (30%)	4 (19%)	1 (16.7%)	4 (44.4%)	1 (33.3%)	3 (37.5%)	2 (28.6%)	0.468
	Medium quality (3.00) n:31	2 (100%)	6 (60%)	11 (52.4%)	2 (33.3%)	3 (33.3%)	2 (66.7%)	4 (50%)	1 (14.3%)	
	High quality (4.00-5.00) n:17	0 (0%)	1 (10%)	6 (28.6%)	3 (50%)	2 (22.2%)	0 (0%)	1 (12.5%)	4 (57.1%)	
Reader’s age	13-14 years old (fifth and sixth graders) n:1 (1.5%)	0 (0%)	0 (0%)	0 (0%)	0 (0%)	0 (0%)	0 (0%)	0 (0%)	1 (14.3%)	0.770
	14-15 years old (sixth and seventh graders) n:4 (6.1%)	0 (0%)	1 (10%)	2 (9.5%)	1 (16.7%)	0 (0%)	0 (0%)	0 (0%)	0 (0%)	
	15-16 years old (eighth and ninth graders) n:2 (3%)	0 (0%)	1 (10%)	0 (0%)	0 (0%)	0 (0%)	0 (0%)	1 (12.5%)	0 (0%)	
	16-17 years old (Ninth to Tenth Graders) n:13 (19.7%)	0 (0%)	0 (0%)	4 (19%)	1 (16.7%)	1 (11.1%)	1 (33.3%)	4 (50%)	2 (28.6%)	
	17-18 years old (tenth to eleventh graders) n:16 (24.2%)	0 (0%)	3 (30%)	4 (19%)	1 (16.7%)	4 (44.4%)	2 (66.7%)	1 (12.5%)	1 (14.3%)	
	18-19 years old (twelfth graders) n:3 (4.5%)	0 (0%)	1 (10%)	1 (4.8%)	1 (16.7%)	0 (0%)	0 (0%)	0 (0%)	0 (0%)	
	19-20 years old (twelfth graders) n:9 (13.6%)	1 (50%)	2 (20%)	3 (14.3%)	0 (0%)	1 (11.1%)	0 (0%)	0 (0%)	2 (28.6%)	
	20-21 years old (college level entry) n:3 (4.5%)	0 (0%)	0 (0%)	1 (4.8%)	0 (0%)	0 (0%)	0 (0%)	1 (12.5%)	1 (14.3%)	
	21-22 years old (college level) n:1 (1.5%)	0 (0%)	1 (10%)	0 (0%)	0 (0%)	0 (0%)	0 (0%)	0 (0%)	0 (0%)	
	College graduate n:14 (21.2%)	1 (50%)	1 (10%)	6 (28.6%)	2 (33.3%)	3 (33.3%)	0 (0%)	1 (12.5%)	0 (0%)	

Reliability of the websites

The JAMA scoring system [7] was used to assess the reliability of the 66 websites included in the study. Inter-rater reliability was found to be excellent for all human-scored metrics (ICC > 0.85). Website typologies were compared in terms of JAMA scores, and no statistically significant difference was observed (p>0.05). Of the websites included in the study, 66.7% received a partial reliability score (2.0-3.0), 30.3% had an insufficient reliability score(0.0-1.0), and 3% received a complete (4.0) JAMA security score (Table 1).

The GQS (low, medium, high) [9] and modified DISCERN scales [10] were used in the quality assessment of the 66 websites included in the study. When the website typologies were compared in terms of GQS and modified DISCERN, no significant difference was observed in both GQS score (p=0.468) and modified DISCERN score (p=0.646). It was determined that news websites received the highest rate of 5.00 modified DISCERN score, with 33.3%. Of the websites included in this study, 25.7% received a GQS score of 4-5. The website type that received the highest GQS score was health-related, with 28.6%, while the lowest GQS score was commercial websites with 28.6% (Table 1).

Readability of the websites

The median values in the readability assessment of the 66 websites included in the study were as follows: FRES 40.81 (difficult), GFOG 14.78 (college level), FKGL 12.18 (12th grade), CL 12.89 (college level), SMOG 12.34 (12th grade), ARI 12.49 (12th grade), LW 13.30 (college level) and GRL 15.25 (college level). The readability values of the contents were compared with the 6th GRL, and all results were significantly higher than the 6th GRL (p <0.01). The types of websites were compared in terms of readability levels, and no significant difference was found (p>0.05).

Correlation analysis 

When the data obtained from the websites included in the study were examined for correlation, none was observed between the scores obtained from the JAMA, DISCERN, and GQS scales and the values obtained from the FRES, GFOG, FKGL, CL, SMOG, ARI, and LW scoring systems. This shows that there is no parallel between the reliability and quality of the websites we included in the study and their easy readability. A low-level negative correlation was found between Blexb rank and JAMA (r=-0.293, p=0.023). This data also indicates that there is a connection between websites with low rank values and reliability. The correlations between Blexb rank and readability scales were as follows: weak negative (p=0.015) correlation with FRES scores; ARI (p=0.015); weak positive correlation with GFOG (p=0.004), FKGL (p=0.004), CL (p=0.008), and SMOG (p=0.004) scores (Table 2).

**Table 2:** Correlation between GQS, JAMA, DISCERN, Blexb, ARI, FRES, GFOG, FKGL, CL and SMOG ** Correlation is significant at the 0.01 level (2-tailed); *Correlation is significant at the 0.05 level (2-tailed) JAMA: Journal of the American Medical Association, DISCERN: Decision Index for Systematic Consumer Evaluation of Reviewed Narratives, GQS: Global Quality Scale, ARI: Automated Readability Index, FRES: Flesch Reading Ease Score, GFOG: Gunning Fog, FKGL: Flesch-Kincaid Grade Level, CL: Coleman-Liau, SMOG:  Simple Measure of Gobbledygook

Spearman's rho		GQScg	JAMAcg	DISCERN	blexb	ARI	FRES	GFOG	FKGL	CL	SMOG
GQS	Correlation coefficient	10.000	0.506^**^	0.599^**^	-0.152	0.181	-0.135	0.150	0.157	0.157	0.150
	Significant (2-tailed)	.	<0.001	<0.001	0.248	0.145	0.280	0.229	0.209	0.208	0.229
JAMA	Correlation coefficient	0.506^**^	10.000	0.686^**^	-0.293^*^	0.207	-0.190	0.149	0.195	0.191	0.183
	Significant (2-tailed)	<0.001	.	<0.001	0.023	0.095	0.126	0.233	0.116	0.125	0.141
DISCERN	Correlation coefficient	0.599^**^	0.686^**^	10.000	-0.125	0.184	-0.235	0.157	0.220	0.232	0.187
	Significant (2-tailed)	<0.001	<0.001	.	0.341	0.140	0.058	0.207	0.076	0.061	0.132
Blexb	Correlation coefficient	-0.152	-0.293^*^	-0.125	10.000	0.312^*^	-0.356^**^	0.371^**^	0.362^**^	0.338^**^	0.364^**^
	Significant (2-tailed)	0.248	0.023	0.341	.	0.015	0.005	0.004	0.004	0.008	0.004
ARI	Correlation coefficient	0.181	0.207	0.184	0.312^*^	10.000	-0.938^**^	0.943^**^	0.980^**^	0.906^**^	0.957^**^
	Significant (2-tailed)	0.145	0.095	0.140	0.015	.	<0.001	<0.001	<0.001	<0.001	<0.001
FRES	Correlation coefficient	-0.135	-0.190	-0.235	-0.356^**^	-0.938^**^	10.000	-0.948^**^	-0.972^**^	-0.969^**^	-0.964^**^
	Significant (2-tailed)	0.280	0.126	0.058	0.005	<0.001	.	<0.001	<0.001	<0.001	<0.001
GFOG	Correlation coefficient	0.150	0.149	0.157	0.371^**^	0.943^**^	-0.948^**^	10.000	0.969^**^	0.885^**^	0.987^**^
	Significant (2-tailed)	0.229	0.233	0.207	0.004	<0.001	<0.001	.	<0.001	<0.001	<0.001
FKGL	Correlation coefficient	0.157	0.195	0.220	0.362^**^	0.980^**^	-0.972^**^	0.969^**^	10.000	0.919^**^	0.982^**^
	Significant (2-tailed)	0.209	0.116	0.076	0.004	<0.001	<0.001	<0.001	.	<0.001	<0.001
CL	Correlation coefficient	0.157	0.191	0.232	0.338^**^	0.906^**^	-0.969^**^	0.885^**^	0.919^**^	10.000	0.906^**^
	Significant (2-tailed)	0.208	0.125	0.061	0.008	<0.001	<0.001	<0.001	<0.001	.	<0.001
SMOG	Correlation coefficient	0.150	0.183	0.187	0.364^**^	0.957^**^	-0.964^**^	0.987^**^	0.982^**^	0.906^**^	10.000
	Significant (2-tailed)	0.229	0.141	0.132	0.004	<0.001	<0.001	<0.001	<0.001	<0.001	.

Content analysis

On evaluating the content of the websites, definition scored 87.9%, pathophysiology 25.8%, epidemiology 27.3%, risk factors 66.7%, symptoms 75.8%, tests 31.8%, diagnosis 39.4%, treatment 71.2%, scoring 7.6%, complications 18.2%, morbidity 21.2%, mortality 6.1%, prevention 71.2%, and prognosis 28.8%. When the content of the websites was analysed according to typologies, there was a significant difference between the websites in terms of only the content of ‘sarcopenia treatment’ (p=0.019). While treatment was mentioned at a rate of 90.5% in health-related websites, there was information about treatment at a rate of 33.3% in government websites (Table 3). When the content was analyzed according to countries, a significant difference was found between the websites that mentioned ‘scoring’ (p=0.046). While 21.4% of the websites from the UK mentioned sarcopenia scoring, none of the websites from the USA (0%) mentioned scoring (Table 4).

**Table 3:** Content analysis according to website typologies A p-value <0.05 was considered statistically significant.

Parameters		Physician	Society/ Professional organization	Health-related website	News (%)	Commercial	Government	Hospital	Other	Total	p-value
Definition	+	1 (50%)	9 (90%)	19 (90.5%)	5 (83.3%)	8 (88.9%)	3 (100%)	6 (75%)	7 (100%)	58 (87.9%)	0.585
	-	1 (50%)	1 (10%)	2 (9.5%)	1 (16.7%)	1 (11.1%)	0 (0%)	2 (25%)	0 (0%)	8 (12.1%)	
Pathophysiology	+	0 (0%)	1 (10%)	7 (33.3%)	1 (16.7%)	3 (33.3%)	0 (0%)	2 (25%)	3 (42.9%)	17 (25.8%)	0.628
	-	2 (100%)	9 (90%)	14 (66.7%)	5 (83.3%)	6 (66.7%)	3 (100%)	6 (75%)	4 (57.1%)	49 (74.2%)	
Epidemiology	+	1 (50%)	1 (10%)	5 (23.8%)	2 (33.3%)	5 (55.6%)	0 (0%)	3 (37.5%)	1 (14.3%)	18 (27.3%)	0.330
	-	1 (50%)	9 (90%)	16 (76.2%)	4 (66.7%)	4 (44.4%)	3 (100%)	5 (62.5%)	6 (85.7%)	48 (72.7%)	
Risk factors	+	1 (50%)	8 (80%)	12 (57.1%)	3 (50%)	9 (100%)	3 (100%)	3 (37.5%)	5 (71.4%)	44 (66.7%)	0.108
	-	1 (50%)	2 (20%)	9 (42.9%)	3 (50%)	0 (0%)	0 (0%)	5 (62.5%)	2 (28.6%)	22 (33.3%)	
Symptoms	+	1 (50%)	9 (90%)	15 (71.4%)	3 (50%)	8 (88.9%)	3 (100%)	6 (75%)	5 (71.4%)	50 (75.8%)	0.530
	-	1 (50%)	1 (10%)	6 (28.6%)	3 (50%)	1 (11.1%)	0 (0%)	2 (25%)	2 (28.6%)	16 (24.2%)	
Tests	+	0 (0%)	3 (30%)	4 (19%)	1 (16.7%)	6 (66.7%)	2 (66.7%)	2 (25%)	3 (42.9%)	21 (31.8%)	0.165
	-	2 (100%)	7 (70%)	17 (81%)	5 (83.3%)	3 (33.3%)	1 (33.3%)	6 (75%)	4 (57.1%)	45 (68.2%)	
Diagnosis	+	0 (0%)	4 (40%)	8 (38.1%)	1 (16.7%)	5 (55.6%)	2 (66.7%)	3 (37.5%)	3 (42.9%)	26 (39.4%)	0.711
	-	2 (100%)	6 (60%)	13 (61.9%)	5 (83.3%)	4 (44.4%)	1 (33.3%)	5 (62.5%)	4 (57.1%)	40 (60.6%)	
Treatment	+	1 (50%)	4 (40%)	19 (90.5%)	3 (50%)	9 (100%)	1 (33.3%)	6 (75%)	4 (57.1%)	47 (71.2%)	0.019
	-	1 (50%)	6 (60%)	2 (9.5%)	3 (50%)	0 (0%)	2 (66.7%)	2 (25%)	3 (42.9%)	19 (28.8%)	
Scores	+	0 (0%)	1 (10%)	3 (14.3%)	0 (0%)	1 (11.1%)	0 (0%)	0 (0%)	0 (0%)	5 (7.6%)	0.811
	-	2 (100%)	9 (90%)	18 (85.7%)	6 (100%)	8 (88.9%)	3 (100%)	8 (100%)	7 (100%)	61 (92.4%)	
Complications	+	1 (50%)	3 (30%)	3 (14.3%)	1 (16.7%)	2 (22.2%)	0 (0%)	1 (12.5%)	1 (14.3%)	12 (18.2%)	0.832
	-	1 (50%)	7 (70%)	18 (85.7%)	5 (83.3%)	7 (77.8%)	3 (100%)	7 (87.5%)	6 (85.7%)	54 (81.8%)	
Morbidity	+	0 (0%)	3 (30%)	3 (14.3%)	2 (33.3%)	3 (33.3%)	0 (0%)	1 (12.5%)	2 (28.6%)	14 (21.2%)	0.742
	-	2 (100%)	7 (70%)	18 (85.7%)	4 (66.7%)	6 (66.7%)	3 (100%)	7 (87.5%)	5 (71.4%)	52 (78.8%)	
Mortality	+	0 (0%)	0 (0%)	0 (0%)	1 (16.7%)	2 (22,2%)	0 (0%)	1 (12,5%)	0 (0%)	4 (6,1%)	0.277
	-	2 (100%)	10 (100%)	21 (100%)	5 (83.3%)	7 (77,8%)	3 (100%)	7 (87,5%)	7 (100%)	62 (93,9%)	
Prevention	+	1 (50%)	5 (50%)	14 (66.7%)	6 (100%)	9 (100%)	2 (66.7%)	5 (62.5%)	5 (71.4%)	47 (71.2%)	0.236
	-	1 (50%)	5 (50%)	7 (33.3%)	0 (0%)	0 (0%)	1 (33.3%)	3 (37.5%)	2 (28.6%)	19 (28.8%)	
Prognosis	+	1 (50%)	1 (10%)	4 (19.0%)	4 (66.7%)	2 (22.2%)	0 (0%)	4 (50%)	3 (42.9%)	19 (28.8%)	0.132
	-	1 (50%)	9 (90%)	17 (81.0%)	2 (33.3%)	7 (77.8%)	3 (1000%)	4 (50%)	4 (57.1%)	47 (71.2%)	

**Table 4:** Website content analysis by country A p-value <0.05 was considered statistically significant.

Parameters		USA	UK	Other	Total	P-value
Definition	+	24 (85.7%)	21 (78.6%)	23 (95.8%)	58 (87.9%)	0.261
	-	4 (14.3%)	3 (21.4%)	1 (4.2%)	8 (12.1%)	
Pathophysiology	+	5 (17.9%)	4 (28.6%)	8 (33.3%)	17 (25.8%)	0.429
	-	23 (83.1%)	10 (71.4%)	16 (66.7%)	49 (74.2%)	
Epidemiology	+	7 (25%)	3 (21.4%)	8 (33.3%)	18 (27.3%)	0.684
	-	21 (75%)	11 (78.6%)	16 (66.7%)	48 (72.7%)	
Risk factors	+	18 (64.3%)	9 (64.3%)	17 (70.8%)	44 (66.7%)	0.863
	-	10 (35.7%)	5 (35.7%)	7 (29.2%)	22 (33.3%)	
Symptoms	+	19 (67.9%)	10 (71.4%)	21 (87.5%)	50 (75.8%)	0.235
	-	9 (32.1%)	4 (28.6%)	3 (12.5%)	16 (24.2%)	
Tests	+	6 (21.4%)	6 (42.9%)	9 (37.5%)	21 (31.8%)	0.281
	-	22 (78.6%)	8 (57.1%)	15 (62.5%)	45 (68.2%)	
Diagnosis	+	8 (28.60%)	7 (50%)	11 (45.8%)	26 (39.4%)	0.294
	-	20 (71.4%)	7 (50%)	13 (54.2%)	40 (60.6%)	
Treatment	+	19 (67.9%)	10 (71.4%)	18 (75%)	47 (71.2%)	0.851
	-	9 (32.10%)	4 (28.6%)	6 (25%)	19 (28.8%)	
Scores	+	0 (0%)	3 (21.4%)	2 (8.3%)	5 (7.6%)	0.046
	-	28 (100%)	11 (78.6%)	22 (91.7%)	61 (92.4%)	
Complications	+	4 (14.3%)	3 (21.4%)	5 (20.8%)	12 (18.2%)	0.779
	-	24 (85.7%)	11 (78.6%)	19 (79.2%)	54 (81.8%)	
Morbidity	+	7 (25%)	3 (21.4%)	4 (16.7%)	14 (21.2%)	0.764
	-	21 (75%)	11 (78.6%)	20 (83.3%)	52 (78.8%)	
Mortality	+	2 (7.1%)	0 (0%)	2 (8.3%)	4 (6.1%)	0.555
	-	26 (92.9%)	14 (100%)	22 (91.7%)	62 (93.9%)	
Prevention	+	21 (75%)	8 (57.1%)	18 (75%)	47 (71.2%)	0.424
	-	7 (25%)	6 (42.9%)	6 (25%)	19 (28.8%)	
Prognosis	+	5 (17.9%)	5 (35.7%)	9 (37.5%)	19 (28.8%)	0.241
	-	23 (82.1%)	9 (64.3%)	15 (62.5%)	47 (71.2%)	

Comparing the typology, readability, quality, reliabilityl, and content of the first 10 websites with others

When analysed according to their content, there was a significant difference between the websites in terms of sarcopenia diagnosis (p=0.032) and screening tests (p=0.038) content. While 60% of the websites in the top 10 mentioned sarcopenia screening tests, this rate was 26.8% for the websites after the top 10. Again, 70% of the websites in the top 10 mentioned sarcopenia diagnosis, while 33.9% of the websites after the top 10 included information about the diagnosis. In terms of reliability, a significant difference was seen between the websites in the top 10 and the others (p=0.003). While 20% of the websites in the top 10 received a full JAMA score, no other website received a full score (0%). As for quality, a significant difference was seen between the websites in the top 10 and the others in terms of GQS score (p=0.025), but no significant difference was seen in terms of the DISCERN scale. While 60% of the top 10 websites received high GQS scores, this rate was 19.6% for the remaining websites. No statistically significant difference was found between the top 10 websites and the other websites in terms of typology and readability (p>0.05) (Table 5).

**Table 5:** Comparing the typology, readability, quality, reliability and content of the first 10 websites with the other remaining websites A p-value <0.05 was considered statistically significant.

Scales and parameters			Top 10 (n=10)	Others (n=56)		Total (n=66)	Comparison of the first 10 websites and the remaining websites according to parameters: p-value	Comparison of the 100 websites per the 6th-grade reading level: p-value
Readability Indexes: Mean±SD	FRES*		45.07 (23.35-50.00)	40.25 (6.10-72.84)		40.81 (6.10-72.84)	0.598	<0.001
	GFOG*		13.95 (11.64-19.21)	14.81 (9.10-22.20)		14.78 (9.10-22.20)	0.304	<0.001
	FKGL*		11.84 (10.00-16.90)	12.23 (7.16-18.58)		12.18 (7.16-18.58)	0.242	<0.001
	CL*		12.83 (11.38-15.13)	12.89 (7.14-18.45)		12.89 (7.14-18.45)	0.872	<0.001
	SMOG*		11.76 (8.89-15.72)	12.40 (7.77-17.65)		12.34 (7.77-17.65)	0.287	<0.001
	ARI*		12.33 (9.89-18.38)	12.49 (7.70-19.64)		12.49 (7.70-19.64)	0.520	<0.001
	LW**		12.33 (8.49-22.68)	13.35 (6.89-25.05)		13.29 (6.89-25.05)	0.241	<0.001
	Grade Level**		17.00 (11.00-23.00)	15.00 (8.00-23.00)		15.25 (8.00 -23.00)	0.383	<0.001
DISCERN: n (%)	1.00		0 (0%)	8 (14.3%)		8 (12.1%)	0.181	
	2.00		2 (20%)	20 (35.7%)		22 (33.3%)		
	3.00		5 (50%)	17 (30.4%)		22 (33.3%)		
	4.00		3 (30%)	6 (10.7%)		9 (13.6%)		
	5.00		0 (0%)	5 (8.9%)		5 (7.6%)		
JAMA: n (%)	Insufficient data		3 (30%)	17 (30.4%)		20 (30.3%)	0.003	
	Partially sufficient data		5 (50%)	39 (69.6%)		44 (66.7%)		
	Completely sufficient data		2 (20%)	0 (0%)		2 (3%)		
GQS: n (%)	Low quality		1 (10%)	17 (30.4%)		18 (27.3%)	0.025	
	Medium quality		3 (30%)	28 (50%)		31 (47%)		
	High quality		6 (60%)	11 (19.6%)		17 (25.8%)		
Typology: n (%)	Physician		0 (0%)	2 (3.6%)		2 (3%)	0.586	
	Society/professional organization		0 (0%)	10 (17.9%)		10 (15.2%)		
	Health-related		4 (40%)	17 (30.4%)		21 (31.8%)		
	News		0 (0%)	6 (10.7%)		6 (9.1%)		
	Commercial		2 (20%)	7 (12.5%)		9 (13.6%)		
	Government		1 (10%)	2 (3.6%)		3 (4.5%)		
	Hospital		1 (10%)	7 (12.5%)		8 (12.1%)		
	Other		2 (20%)	5 (8.9%)		7 (10.6%)		
	Content		n (%)	n (%)		n (%)		
	Definition	+	10 (100%)	48 (85.7%)	58 (87.9%)		0.202	
		-	0 (0%)	8 (14.3%)	8 (12.1%)			
	Pathophysiology	+	2 (20%)	15 (26.8 %)	17 (25.8%)		0.651	
		-	8 (80%)	41 (73.2%)	49 (74.2%)			
	Epidemiology	+	2 (20%)	16 (28.6%)	18 (27.3%)		0.575	
		-	8 (80%)	40 (71.4%)	48 (72.7%)			
	Risk Factors	+	9 (90%)	35 (62.5%)	44 (66.7%)		0.089	
		-	1 (10%)	21 (32.5%)	22 (33.3%)			
	Symptoms	+	10 (100%)	40 (71.4%)	50 (75.8%)		0.052	
		-	0 (0%)	16 (28.6%)	16 (24.2%)			
	Tests	+	6 (60%)	15 (26.8%)	21 (31.8%)		0.038	
		-	4 (40%)	41 (73.2%)	45 (68.2%)			
	Diagnosis	+	7 (70%)	19 (33.9%)	26 (39.4%)		0.032	
		-	3 (30%)	37 (66.1%)	40 (60.6%)			
	Treatment	+	8 (80%)	39 (69.6%)	47 (71.2%)		0.505	
		-	2 (20%)	17 (30.4%)	19 (28.8%)			
	Scores	+	0 (0%)	5 (8.9%)	5 (7.6%)		0.326	
		-	10 (100%)	51 (91.1%)	61 (92.4%)			
	Complications	+	0 (0%)	12 (21.4%)	12 (18.2%)		0.106	
		-	10 (100%)	44 (78.6%)	54 (81.8%)			
	Morbidity	+	3 (30%)	11 (19.6%)	14 (21.2%)		0.461	
		-	7 (70%)	45 (80.4%)	52 (78.8%)			
	Mortality	+	0 (0%)	4 (7.1%)	4 (6.1%)		0.383	
		-	10 (100%)	51 (92.8%)	62 (93.9%)			
	Prevention	+	6 (60%)	41 (73.2%)	47 (71.2%)		0.395	
		-	4 (40%)	15 (26.8%)	19 (28.8%)			
	Prognosis	+	2 (20%)	17 (30.4%)	19 (28.8%)		0.505	
		-	8 (80%)	39 (69.6%)	47 (71.2%)			

## Discussion

More than four billion people use the Internet around the world, with more than 1.9 billion different websites, with a trillion searches per day [22]. As the Internet is an important health information resource for people, health-related problems are the second most common website searched on Google [23]. People usually search for symptoms and treatment options on websites [24].

To our knowledge, this study is the first to evaluate the quality and readability (66 out of 278 website links) of sarcopenia-directed health information on the Internet. Our results showed that most of the websites (66.7%) received a partial JAMA score, which assesses the reliability. All of the websites we had searched had a readability score above 6th GRL, which is hard to understand for the public who are trying to search the Internet for their health problems. The National Institutes of Health suggested a 6th-grade reading level for the average person [21]. Jayasinghe et al. arrived at low readability scores and suggested that simple information would be easier for people to interpret the websites [25]. People prefer simple words and short sentences to clearly understand the information they are seeking.

After eliminating 278 websites, we evaluated 66 websites where definition, risk factors, symptoms, treatment, and prevention were the most mentioned issues about sarcopenia. Government websites mentioned treatment significantly less than the other websites, and USA websites did not mention sarcopenia scoring. This shows that government websites do not give emphasis to treatment.

We searched the internet and compared the first 10 websites in terms of typology, readability, quality, reliability, and content with the other websites. Top 10 websites mentioned screening tests and diagnosis significantly higher than the rest. Also, we realized that the top 10 websites received full JAMA scores and high GQS scores, which are significantly higher than the others. This showed us that people want to learn more about diagnosis and which tests should be done to diagnose the condition. People might decide which hospital to seek treatment at, as private hospitals are more expensive than government hospitals. There was no statistically significant difference between the top 10 and the remaining websites in terms of typology and readability.

In Bagcier et al. and Kocyigit et al.’s studies, they did not find any significant difference between the first 10 and the other websites about readability [26,27]. The first 10 websites should have a reading level lower than 6th GRL to enable the reception of information more accurately, clearly, and quickly. The quality of the websites was similar in terms of GQS and DISCERN [5].

We evaluated the website typologies, with the most common one being health-related websites, followed by commercial websites. But there was no significant difference in readability between them. The readability of the websites in this study is above a 6th-grade reading level. Although the included websites had a reliability of 2-3 JAMA score, the average educated person may not interpret what they are seeking to learn from these websites [8].

In Gu et al.’s systematic review [28] on breast cancer online patient education materials (OPEMs), the average readability was found to be nearly twice the recommended 6th GRL. Gulbrandsen et al. [29] on shoulder arthroscopy and Gao et al. [30] on carpal tunnel syndrome also reported consistently poor readability, with few resources meeting the 6th-grade standard, reinforcing that the excessive complexity is a systemic issue across diverse health topics, not unique to sarcopenia. Similarly, our findings, with a median FRES of 40.81, align with low scores reported in other musculoskeletal and chronic conditions, such as plantar fasciitis (mean FRES score of 42.6) [8], and carpal tunnel syndrome (mean readability range of 10.8-15.3) [31]. This consistent pattern across the literature underscores the critical need for immediate intervention in online health information development.

In Bhatt et al.’s study [32], the mean readability score of cholesterol education materials was approximated at an 11th-grade reading level, which exceeded the health literacy level of the average North American adult. Like our study, they also concluded that patient materials should be maximum 6th-grade reading level. They advised that the improvement of the readability and understandability of educational materials may result in a greater likelihood of improving healthy behavioral changes of lay people.

Varli et al. [33] evaluated the readability of PEM on female pelvic floor disorders. Women hesitate to seek help from healthcare providers as they find it difficult to share complaints of involuntary leakage or vaginal prolapse. As a result, they often look toward websites. The readability level of Turkish education materials on the websites of national or international authorities is also above the recommended 6th GRL. Hanci [6] aimed to investigate the readability of Internet-based PEM related to *Helicobacter pylori *infection and found the readability level to be significantly higher than the recommended 6th GRL. Hanci concluded that the quality and reliability of patient education materials are low to moderate. Unfortunately, all studies, including ours, lack a suitable reading level of the general population.

All educational websites should have a 6th-grade readability level. Websites need to simplify their sentences, optimize their vocabulary, and use simple, patient-friendly sentences to explain the topic. Improving the clarity of information will increase patient health literacy and ultimately improve patient health care.

Considering that sarcopenia is a progressive condition associated with frailty, falls, hospitalization, and increased mortality, the lack of accessible and comprehensible online information may pose a barrier to timely diagnosis and treatment initiation. From a clinical perspective, this gap may indirectly influence patient engagement, as individuals with insufficient knowledge might be less likely to seek medical evaluation or adhere to preventive strategies. Therefore, improving the quality and readability of online sarcopenia materials is hypothesized to have the potential to enhance early detection, patient engagement, and ultimately reduce the disease burden in clinical practice. We acknowledge that this study, being cross-sectional and not patient-assessed, cannot establish a direct causal link between poor readability and adverse patient outcomes.

Limitations

We acknowledge several limitations inherent to this study design. First, the cross-sectional nature of the analysis means our findings represent a snapshot in time (August 2024) and may not reflect continuous changes in online content or search engine algorithms. Second, the most important limitation is that we only included texts in English. Similar studies should be conducted for other widely used languages. Third, although we used validated indices, readability formulas provide proxies for comprehension and do not capture whether real patients truly understand and act on the information. Future studies should incorporate patient-centered validation methods (e.g., usability testing or the teach-back method). Fourth, despite addressing subjectivity by reporting a high ICC, the initial content classification requires human judgment. Finally, we examined a total of 278 results, of which only 66 websites were suitable for evaluation. Although we used the same limitation criteria as other studies, we determined that the content providing information to the public was less about sarcopenia. Therefore, there is a lack of sufficient sites providing information to the public about sarcopenia. Quality websites that are readable and aimed at informing the public about sarcopenia should be increased.

Strengths

This study included websites used by people with an average education level, and academic contents were excluded from the study. We sourced 278 websites, a number that is quite high compared to other studies. It constitutes a wide representation of online sarcopenia information materials. Another strength of this study is that, to the best of our knowledge, it is the first and only study currently evaluating the readability of PEM related to sarcopenia.

## Conclusions

Sarcopenia online PEM consistently demonstrated readability levels significantly above the recommended 6th-grade reading level. Although overall website quality was low-to-moderate, a key finding was the significant difference in reliability and quality of the top 10 ranked websites, which achieved full JAMA scores (reliability) and higher GQS scores (quality) compared to the rest (p=0.003 and p=0.025, respectively). It is important to note that no significant correlation was found between general readability metrics (e.g., FRES, SMOG) and website quality/reliability metrics (JAMA, DISCERN, GQS). The content analysis highlighted health portals as the best-performing category and revealed a concerning lack of information on scoring/screening tests. Developing simpler, more reliable, and easily accessible online materials is critical to enable patients to recognize risk factors and symptoms earlier, thereby improving the overall management of sarcopenia.
